# Unusual association between testicular tuberculosis and microdeletion of the Y chromosome in an infertile patient with azoospermia

**DOI:** 10.1016/j.amsu.2022.104068

**Published:** 2022-06-25

**Authors:** Moez Rahoui, Yassine Ouanes, Kays Chaker, Mokhtar Bibi, kheireddine Mourad Daly, Ahmed Sellami, Sami Ben Rhouma, Yassine Nouira

**Affiliations:** Urology Department La Rabta Hospital, Tunis, Tunisia

**Keywords:** Infertility, Azoospermia, Testicular tuberculosis, Y chromosome microdeletion

## Abstract

**Introduction:**

and importance: Infertility affects approximately 10–15% of couples worldwide. Several causes are incriminated such as hormonal abnormalities, infections, genetic disorders, testicular cancer, varicocele, and others. Herein, we report a case of an unusual association between testicular tuberculosis and microdeletion of the Y chromosome in an infertile patient and we discuss the diagnostic and therapeutic difficulties.

**Case presentation:**

A 36-year-old patient, a smoker, with no previous history consulted our department for primary infertility for 2 years. The clinical examination was normal. The sperm count showed azoospermia. karyotype analysis confirmed the diagnosis of a microdeletion of the Y chromosome. A testicular biopsy was performed. The microscopic analysis did not find any sperm cells. However, the histopathological examination was in favor of testicular TB. The patient received 6 months of anti-TB treatment. He remained azoospermic.

**Clinical discussion:**

Azoospermia is defined as the absence of sperm in the ejaculate in two different samples. This condition is classified as obstructive and non-obstructive. The etiology of this condition is either an intrinsic testicular deficiency or an insufficient production of gonadotropins. Genetic and chromosomal abnormalities should be investigated due to the higher incidence in azoospermic patients compared to the normal population. Testicular causes are dominated by infections, trauma, ischemia, and iatrogenic causes such as chemotherapy and radiotherapy. Genetic causes are dominated by Klinefelter syndrome and Y-chromosome microdeletions.

**Conclusion:**

Azoospermia is a frequent cause of male infertility. Several causes are incriminated such as hormonal abnormalities, infections, genetic disorders, and others. In some cases, this condition can be multifactorial.

## Highlights

• Infertility affects approximately 10–15% of couples worldwide.
• Several causes are incriminated such as hormonal abnormalities, infections, genetic disorders, and others.
• In some cases, this condition can be multifactorial.
• We report a case of an unusual association between a microdeletion of the Y chromosome and testicular tuberculosis.

## 1 Introduction

Infertility is defined as the inability of a couple to conceive a child after at least one year of unprotected sex [1]. This problem affects about 10–15% of married couples, and the male factor is responsible for half of the cases [1]. Several causes are incriminated such as hormonal abnormalities, infections, genetic disorders, testicular cancer, varicocele, and others [2]. Testicular tuberculosis is a rare cause of male infertility in underdeveloped countries [3]. In addition, Y chromosome microdeletions are the most frequent genetic cause of male infertility after Klinefelter's syndrome [2]. To our knowledge, there are no cases reported in the literature concerning the association between testicular tuberculosis (TB) and microdeletion of the Y chromosome. The aim of this case was to discuss the diagnostic and therapeutic difficulties of this unusual association. **The work has been reported in line with the SCARE 2020 criteria** [4]**.**

## 2 Case report

**Fig. 1:**
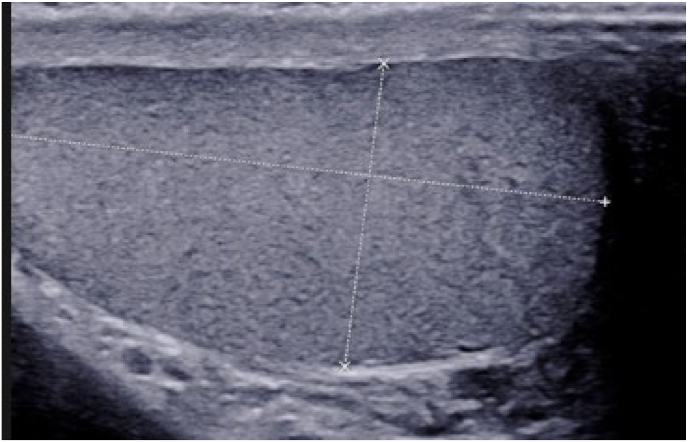
Testicular ultrasound showing a normal appearance of the testicles.

**Fig. 2:**
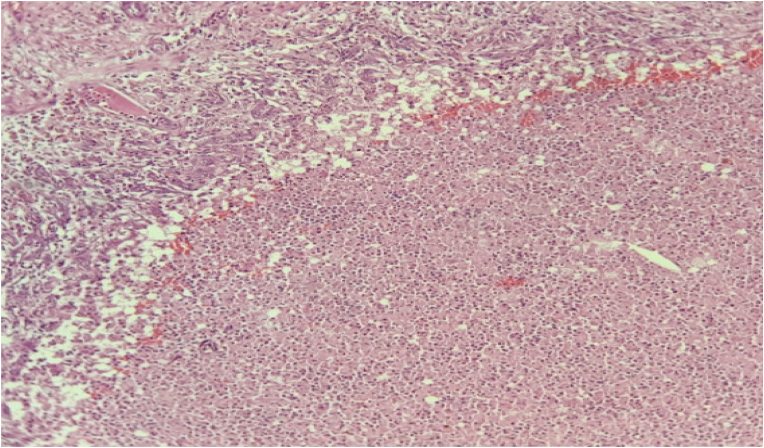
Testicular parenchyma seat granuloma epithelioid and giant cell centered by caseous necrosis (hematoxylin and eosin × 40**)**.

A 36-year-old patient, a smoker, with no previous history consulted our department for primary infertility for 2 years. He has been married for 2 years; his 28-year-old wife was in good condition. The urological examination was normal with two testicles of normal size. The patient had no erectile dysfunction. Testicular ultrasound showed two normal-sized testicles, with no suspicious lesions. There was no varicocele (Fig. 1). The sperm count showed azoospermia which was confirmed after a second sample taken after 3 months. To determine the type of azoospermia, a hormonal analysis was performed. The hormonal exploration showed an elevated FSH level, an elevated LH level, a normal prolactin level, and a low testosterone level (Table 1). The secretory etiology of the azoospermia was retained and the patient was referred to the genetic department for exploration. karyotype analysis confirmed the diagnosis of a microdeletion of the Y chromosome. A testicular biopsy was performed. The microscopic analysis did not find any sperm cells. However, the histopathological examination was in favor of testicular TB (caseous necrosis and Langhans giant cell isolated) (Fig. 2). Based on the histological findings, we sent the biopsy specimen for bacteriological analysis. Tuberculosis-polymerase chain reaction (TB-PCR) testing confirmed the diagnosis of TB disease by confirming the presence of the *Mycobacterium tuberculosis* complex. The diagnosis of renal tuberculosis was retained.

**Table 1:** Semen analysis results and hormonal analysis.

Variables	Value	Normal value
**Semen analysis**		
Appearance	Normal	
Viscosity	Normal	
Volume	2.5	>2
PH	7.5	7.2–7.8
Sperm count:	0	>20 million/ml
WBCs	15–20	0-2/HPF
**FSH level**	14,75	1-12 UI/ml
**LH level**	13,24	2-12 UI/ml
**Prolactin level**	15,12	17 ng/ml
**Testosterone level**	130	270–1070 ng/dl

We performed an intravenous urography examination without finding any abnormalities in other structures of the urinary tract. The patient received 6 months of anti-TB treatment. Four antibiotics (rifampicin, isoniazid, pyrazinamide, and ethambutol) for 2 months followed by two antibiotics (isoniazid and rifampicin) for 4 months. After the treatment, a semen analysis was performed. The patient remained azoospermic.

## 3 Discussion

Azoospermia is defined as the absence of sperm in the ejaculate in two different samples. It inevitably leads to infertility [1]. Worldwide, it is estimated that 1% of men of reproductive age and up to 10% of men with infertility are azoospermic [2]. Azoospermia is classified as obstructive and non-obstructive [2,3]. This differentiation is clinically significant because it affects patient management and treatment outcomes.

Non-obstructive azoospermia reflects testicular insufficiency due to impaired spermatogenesis [2,3]. Its etiology is either an intrinsic testicular deficiency or an insufficient production of gonadotropins. Genetic and chromosomal abnormalities should be investigated due to the higher incidence in azoospermic patients compared to the normal population [2]. Testicular causes are dominated by infections, trauma, ischemia, and iatrogenic causes such as chemotherapy and radiotherapy [5].

Genetic causes are dominated by Klinefelter syndrome and Y-chromosome microdeletions [2]. The relationship between azoospermia and Y chromosome deletions was first described by Tiepolo in 1976 [5]. The incidence of this genetic abnormality is 10–15% in azoospermic patients [2]. It is a condition that is transmitted from father to son with variable penetrance. It can be responsible for severe oligospermia or azoospermia. Microdeletions of the Y chromosome are located on the long arm. They affect the AZF region inducing an alteration of spermatogenesis [5]. On hormonal analysis, there is peripheric hypogonadism with elevated FSH and LH and low testosterone [5]. Genetic diagnosis of Y chromosome microdeletions is based on the polymerase chain reaction (PCR) technique using AZF markers [2,5]. The fertility prognosis of the patient depends on the type of microdeletion. Accumulating studies demonstrate that the deletion of AZFa, AZFb, and AZFc are the most common genetic microdeletions in Y chromosome for infertile male throughout the world [1,2,5]. It is reported that the deletion of AZFa and AZFb in the Y chromosome portends an exceptionally poor prognosis for sperm retrieval [5]. Therefore, the assisted productive treatment of intracytoplasmic sperm injection is only recommended for patients with partial AZFb and AZFc microdeletion [5]. A testicular biopsy allows the search for spermatozoa in the seminiferous tubules.

In some cases, azoospermia can be multifactorial, associating genetic anomalies with other causes, notably infectious, hormonal or iatrogenic [2]. Our patient, with azoospermia secondary to microdeletion of the Y chromosome, was diagnosed with testicular tuberculosis by biopsy.

Genital TB is uncommon, and testicular TB is further rare, comprising only 3% of genital TB [3]. It is usually seen in the course of disseminated tuberculosis, but isolated testicular tuberculosis is extremely rare [3]. Clinically, testicular TB may mimic other testicular lesions, such as a testicular tumor, infarction, or even testicular torsion. Clinically, testicular tuberculosis may mimic other testicular lesions, such as a testicular tumor, infarction, or even testicular torsion [6]. It usually affects men between the ages of 20 and 40. The clinical examination may reveal painless or painful scrotal swelling or scrotal fistulas. Diagnosis is based on testicular ultrasound and bacteriological sampling [3]. In some cases, testicular biopsy with histological examination allows the positive diagnosis of this pathology [6]. Recently, nucleic acid amplification techniques, such as PCR, have been widely studied for the detection of *M. tuberculosis* and other mycobacteria [3]. In our patient, the diagnosis was suspected on histological findings and was confirmed by tuberculosis-polymerase chain reaction (TB-PCR) testing.

In the literature, the diagnosis of testicular tuberculosis was often made with a scrotal mass [6,7]. The diagnosis was confirmed by histological examination after orchiectomy. This condition can cause an alteration of spermatogenesis reaching its total stop inducing infertility. The treatment is based on anti-tuberculosis chemotherapy including rifampicin, isoniazid, pyrazinamide, and ethambutol [3,6,7]. The fertility prognosis is correlated with the severity of cellular damage, which may be irreversible. Gbessi et al. reported a case of azoospermia secondary to testicular tuberculosis. The patient remained azoospermic despite orchiectomy and well-conducted anti-tubercular treatment [3].

In the reported case, our patient was put under anti-tuberculosis treatment for 6 months. He remained azoospermic after the treatment.

## 4 Conclusion

Azoospermia is a frequent cause of male infertility. Several causes are incriminated such as hormonal abnormalities, infections, genetic disorders, and others. In some cases, this condition can be multifactorial. We report a case of an unusual association between a microdeletion of the Y chromosome and testicular tuberculosis in an infertile man with azoospermia.

## Ethical approval

Not applicable.

## Sources of funding

This research did not receive any specific grant from funding agencies in the public, commercial, or not-for-profit sectors.

## Author contribution

Rahoui Moez, Ouannes Yassine and Chaker Kays: Data collection, Manuscript writing, Results discussion. Bibi Mokhtar, Mourad Daly Kheireddine and Sellami Ahmed: Manuscript writing and revision. Ben Rhouma Sami and Nouira Yassine: Paper revision.

## Guarantor

Rahoui moez is the guarantor of the study and accept full responsibility for the work and/or the conduct of the study, had access to the data and controlled the decision to publish.

## Registration of research studies

1. Name of the registry:: N/a
2. Unique identifying number or registration ID:: N/a
3. Hyperlink to your specific registration (must be publicly accessible and will be checked):: N/a

## Consent

Written informed consent was obtained from the patient for publication of this case report and accompanying images. A copy of the written consent is available for review by the Editor-in-Chief of this journal on request.

## Provenance and peer review

Not commissioned, externally peer-reviewed.

## Declaration of competing interest

Authors do not report any conflict of interest.
